# Comparing the Ability of Various Resin-Based Composites and Techniques to Seal Margins in Class-II Cavities

**DOI:** 10.3390/polym13172921

**Published:** 2021-08-30

**Authors:** Abdullah Saleh Aljamhan, Sultan Ali Alhazzaa, Abdulrahman Hamoud Albakr, Syed Rashid Habib, Muhammad Sohail Zafar

**Affiliations:** 1 Restorative Dental Sciences Department, College of Dentistry, King Saud University, Riyadh 11545, Saudi Arabia; a.aljamhan@ksu.edu.sa; 2 Security Aviation, Riyadh 11622, Saudi Arabia; hz.sultan9@gmail.com; 3 Prince Sultan Military Medical City, Riyadh 11543, Saudi Arabia; ah.albakr@gmail.com; 4 Department of Prosthetic Dental Sciences, College of Dentistry, King Saud University, Riyadh 11545, Saudi Arabia; 5 Department of Restorative Dentistry, College of Dentistry, Taibah University, Al Madinah Al Munawwarah 41311, Saudi Arabia; MZAFAR@taibahu.edu.sa; 6 Department of Dental Materials, Islamic International Dental College, Riphah International University, Islamabad 44000, Pakistan

**Keywords:** adhesives, dentin, RBCs, restorations, micro-leakage, class-II cavities, restorative materials

## Abstract

*Background:* Resin-based composites (RBCs) provide excellent esthetics but the marginal micro-leakage in the proximal cavities remains a major concern. The aim of the present study was to assess the ability of various dental RBCs and techniques utilized for sealing deep dentin margin in class-II cavities. *Methods:* Box-cavities (class-II) on the distal and mesial surfaces of extracted (premolar) teeth were prepared with a gingival margin placed 1mm apical to the cemento-enamel junction. Teeth with prepared class II cavities were randomly divided into four study groups according to the type of restorative materials (conventional RBC; bulk-fill RBC; conventional RBC lined with flowable RBC and conventional RBC lined with resin-modified glass-ionomer-cement (GIC) as open sandwich-technique). Each group was further subdivided into a total-etch subgroup in which a separate etching step was performed before applying the bonding agent and a self-etch subgroup in which a self-etch adhesive system was used (*n* = 10). For each group, cavities were restored using the respective restorative materials and techniques, subjected to 1000 thermocycles, and placed in the methylene-blue dye. The specimen teeth were sectioned for further microscopic examination for micro-leakage. *Results:* The least dye penetration values were reported for group 4 (GIC) followed by the group Bulk-fill using the self-etch adhesive system (group 2b). The highest dye penetration was reported for the group Bulk-fill using the total-etch adhesive system (2a), followed by the group conventional RBC using the total-etch adhesive system). The total-etch adhesive system had significantly greater micro-leakage compared to the self-etch adhesive system (1a) (*p* = 0.026). *Conclusions:* The self-etch adhesive system significantly reduced the micro-leakage compared to the total-etch system. Bulk-fill RBC when bonded with the self-etch adhesive provided good marginal sealing ability comparable to open sandwich-technique using GIC.

## 1. Introduction

In recent years, patient awareness toward cosmetic restorations has increased, therefore, tooth-colored restorations have become the most demanding restorations both in anterior as well as posterior regions of the oral cavity. In terms of esthetics, adhesive properties with smaller cavity size and improvement in the strength of remaining tooth structure, RBCs restorations are better as compared to conventionally used amalgam restorations. Unlike amalgam restorations, RBCs do not need retention and resistance form and are considered suitable material for direct restorations [1,2]. However, the longevity of the RBCs restorations remains a major concern. Compared to the amalgam restorations, the failure rate of RBCs is higher in terms of postoperative sensitivity and caries (secondary) mainly caused by micro-leakage especially at the cervical margin of deep cavities [3,4,5]. Micro-leakage is, “the penetration of bacteria, fluids, molecules, or ions into the spaces between the cavity walls and the restorative materials, resulting in sensitivity, recurrent caries, discoloration of the restoration margins, irritation of the pulp, and restoration failure” [6]. To overcome the problem of micro-leakage and to fulfill the increase in patient’s demand toward a cost-effective, biocompatible, esthetic, and durable dental restoration has encouraged the researchers to carry out research for improving effectiveness/longevity of dental RBCs adhesive bonds to the tooth structure, predominantly at the gingival margins of class-II cavities [7].

Various RBCs materials, adhesive systems/techniques and placement techniques have been employed for improving the reliability of restorations clinically and controlling the contraction-stresses due to polymerization of the resin [8,9,10]. The adhesive bonds between the RBCs materials and the tooth structure are greatly affected by the shrinkage stresses developed at the tooth-restoration interface resulting in compromised bond and marginal sealing of these adhesive restorations. Complete adaptation of these adhesive restorations to the cavity margins is essential for long-term success of these restorations. The combined glass ionomer and RBCs restoration, known as the sandwich technique, provides a reliable chemical bond to the dentin, micromechanical bonding of the RBCs to the glass ionomer surface and an acceptable esthetic result. [6,7].

In recent years Bulk-fill resin has been introduced into clinical practice [11,12]. SonicFill is a resin-based direct dental restorative material that has low shrinkage. It is indicated for direct placement in all cavity classes in anterior and posterior teeth. It has several advantages compared to incrementally placed RBCs. For example, it is fast and easy to place. Also, it flows easily and has good consistency after placement, good polishability, and excellent radiopacity. However, it has some limitations which require a handpiece for the placement of RBCs, learning curve to avoid incorporating voids, and overfilling/contouring the tip too large for some minimal preparation [13]. Manufacturer and few reports claim there is a reduction in polymerization shrinkage stress, reduced cuspal deflection during light irradiation compared to the conventionally cured RBCs, and comparable curing efficiency at 4 mm [9,14]. Greater depth of curing and placement in bulk increments of up to 4 mm with complete polymerization are few more advantages. In addition, the “stress decreasing resin technology” was adopted for minimizing the shrinkage stress and to allow the placement of material in bulk. This would help clinically to eliminate the need for the placement of materials in increments for curing and decrease the need for RBCs manipulation during placement [10]. The incorporation of nanotechnology has improved the properties of RBC dental restoratives [15,16,17]. SonicFill (nanohybrid bulk fill RBC) utilizes a sonically activated handpiece, attached to the high-speed multiflex connection for placement of material in a flowable state. Activation of the handpiece results in lowering the viscosity of the material, which helps in extruding the RBC that had had non-flowable consistency before [13]. 

Advancements in adhesive systems and adhesion, have led to the development of numerous adhesive systems which are available commercially. The Etch and Rinse (ER); Total-etch (TE); Self- Etch (SE) and glass-ionomers are the most popular adhesive systems [18,19,20]. The ER adhesives involve the application of phosphoric acid, permitting the demineralization of the dental tissues, and thorough removal of the smear layer after rinsing [21]. Therefore, in the process of ER, adhesive resin bonding is carried out for different clinical procedures resulting in the demineralization and the hybridization of dental structures repeatedly. The objective of this experimental in vitro research was to investigate the ability of various RBCs adhesive restorative materials and techniques used to seal deep dentin margins in class-II cavities. The null hypothesis was that there will be no difference in micro-leakage score between Bulk-fill RBCs and other restorative materials. In addition, we hypothesized that there is no difference in the efficiency of self-etch adhesives and total-etch adhesives in sealing dentin margin.

## 2. Materials and Methods

### 2.1. Sample Collection and Storage

Freshly extracted intact human premolar teeth (*n* = 40) were collected. After collection, all the teeth were inspected to assure that there were no caries. Teeth with caries, previous restorations, cracks, stains, or root-canal treated were excluded. Collected teeth were stored in normal saline. The teeth were cleaned/washed with normal saline and examined under a stereomicroscope for excluding the teeth with micro-cracks/defects.

### 2.2. Preparation of Teeth

Box cavities (class-II) were prepared on the mesial and distal sides with a #330 pear-shaped carbide bur (Brasseler Dental, LLC, One Brasseler Blvd. Savannah, GA, USA) using high-speed air/water-cooled turbine. To ensure cutting efficiency, a new bur was used for every five preparations. The bucco-lingual and mesio-distal extension of the cavities was 3 mm and 1.5 mm, respectively. The gingival margin was designed approximately 1 mm apical to cemento-enamel junction. All the internal line angles were rounded, cavo-surface margins were left sharp with no bevel and finishing was completed using gingival margin trimmers.

### 2.3. Application of Restorative Materials

Toffelmire metal matrix-band was attached to retainer (Tofflemire Retainer-Universal, Dentsply) and used for each tooth during placement of the material. The matrix was held by finger pressure against the gingival margin of the cavity and was tightened to prevent restoration overhang. The specimen teeth were divided randomly into four main groups, according to restorative material to be tested in the experiment then further subdivided according to the bonding technique, self-etch or total-etch (*n* = 10). The cavities were divided and restored as following;

- Group 1a (control group): Used conventional RBC and total-etch adhesive system.
- Group 1b: Used conventional RBC and self-etch adhesive system.
- Group 2a: Using Bulk-fill RBC with a total-etch adhesive system.
- Group 2b: Using Bulk-fill RBC with a self-etch adhesive system.
- Group 3a: Used conventional and flowable RBC base along with a total-etch adhesive system.
- Group 3b: Conventional and flowable RBC base was applied using a self-etch dentin adhesive system.
- Group 4: Resin-modified GIC base was used under conventional RBC as open sandwich-technique.

All the materials used in the study are presented in Table 1. The tested materials were used/manipulated according to the instructions by the manufacturers.

**Figure 1:**
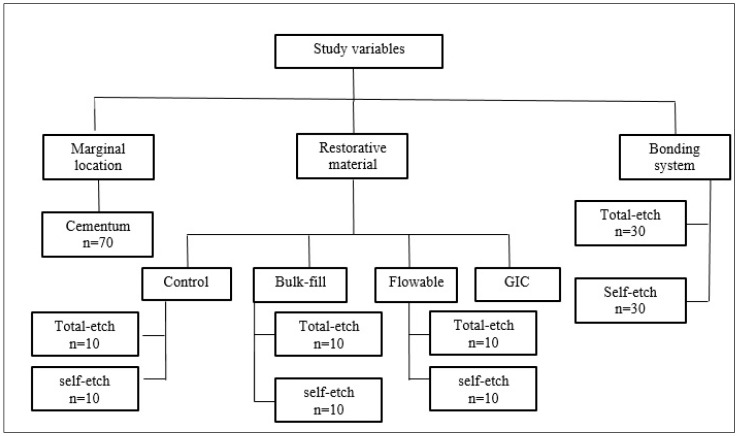
Schematic presentation of the study design and variables.

In Table 2, a summary of the different bonding techniques is listed. For a complete cure of adhesives and restorative materials, a high-intensity LED curing light (3M ESPE Elipar™ S10) was used. The light intensity ranged between 400 to 515 nm with 1200 mW/cm^2^ −10%/+20%, a utilizable wavelength range of 430–480 nm, and wavelength peak of 455 nm +/− 10 nm. Study variables are displayed in (Figure 1).

### 2.4. Thermocycling and Micro-Leakage Test

**Figure 2:**
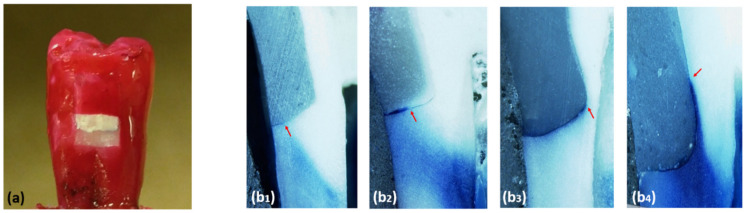
Varnish used to coat all tooth surfaces except 1mm around the gingival margin (**a**). Samples of specimens with different microleakage scores, scores 1, score 2, score 3, score 4 ((**b1**–**b4**) respectively).

Thermocycling of the specimens was carried out as follows: 1000 thermo cycles, 5 °C and 55 °C, 30 s dwell time at each temperature, exchange time of 13s between baths (Huber, SD Mechatronik Thermocycler, D-83620 Feldkirchen-Westerham, Germany). After the thermocycling, the apex of each specimen tooth was sealed with autopolymerizing resin with two coats of fingernail varnish for coating all tooth surfaces except 1mm around the gingival margin (Figure 2). Specimens then were immersed in 0.5% methylene-blue dye. After 24 h of immersing in the dye, the specimens were rinsed under tap water then embedded completely in a self-cure acrylic resin. The teeth were sectioned longitudinally in a mesio-distal direction towards the center of the restorations using a saw (Isomet Precision saw, Lake Bluff, IL, USA). The specimens were analyzed under ×50 magnification in a stereomicroscope by three operators (Figure 2).

The degree of dye penetration was scored according to the following criteria:

0: No penetration.
1: Dye penetrates less than half of the gingival wall.
2: Dye penetrates up to half of the gingival.
3: Dye penetrates more than half of the gingival wall.
4: Dye penetrates the whole length of the gingival wall and along the axial wall.

### 2.5. Statistical Analysis

All the collected data were tabulated and analyzed using SPSS analysis software (SPSS, Inc., Chicago, IL, USA). The Kruskal–Wallis test was used for assessment of the micro-leakage score differences between groups. For the assessment of the pairwise differences among the groups, the Mann–Whitney U-test was utilized.

## 3. Results

**Figure 3:**
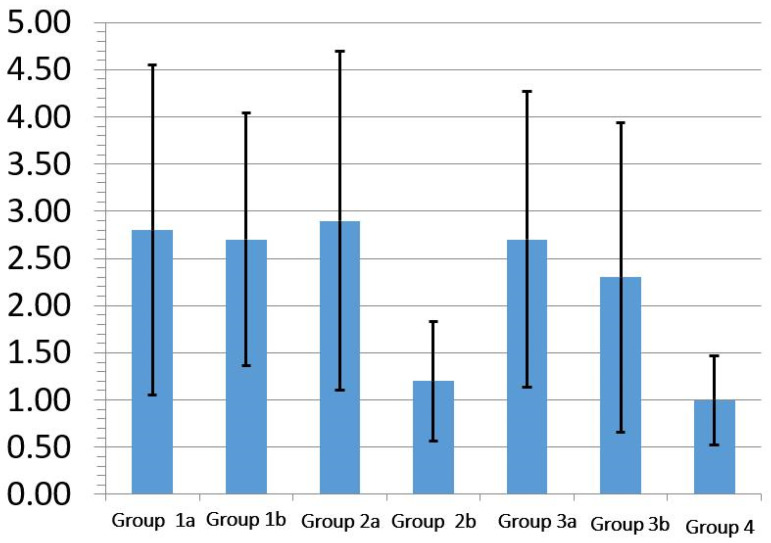
The mean and standard deviation of micro-leakage score for the different tested groups. For each group, the fraction values were obtained from the mean of microleakage scores.

The mean and standard deviation of the micro-leakage score for the different groups are shown in Figure 3. The Kruskal–Wallis Test shows a significant micro-leakage difference between different groups (*p* = 0.021).

The glass-ionomers group shows the minimum micro-leakage score among all the groups. There was no significant difference between the glass-ionomers group and the Bulkfil RBC group when used with self-etch adhesive system (*p* = 0.39) Adding a flowable RBC liner under regular RBC reduces the micro-leakage compared to regular RBC without liner, but the difference was not significant (*p* = 0.59). Table 3 shows the mean micro-leakage score for the total-etch and self-etch adhesive systems. The Mann–Whitney Test shows that the total-etch adhesive system has significantly higher micro-leakage compared to the self-etch adhesive system (*p* = 0.026) (Table 4).

## 4. Discussion

The present study investigated the ability of various polymeric dental adhesives and techniques in terms of sealing deep margins in class-II cavities. For investigating the sealing performance of different restorative materials/techniques, the oral cavity’s environment should be applied. Therefore, we used natural premolar teeth freshly extracted for orthodontic treatment and were disease-free otherwise. Class-II cavities were prepared mimicking the clinical cavity preparation process and subjected to thermocycling in vitro to expose the restoration and the teeth to temperature extremes simulating the introduction of hot and cold temperatures of the oral cavity. The thermocycling treatment might highlight the differences in coefficients of thermal expansion between the teeth tissues and restorative materials, which may be the cause for gap formation [22]. The null hypothesis is partially accepted, as Bulk-fill RBCs using SE demonstrated no significantly higher dye penetration than the open-sandwich technique. However, the null hypothesis was partly rejected as Bulk-fill RBCs using SE demonstrated significantly less dye penetration than conventional incremental fill. The null hypothesis has to be accepted as Bulk-fill RBCs using TE showed no significant difference in micro-leakage score between Bulk-fill RBCs using TE and other restorative materials Moreover, the null hypothesis has to be rejected, as SE showed significantly less dye penetration than TE. According to the majority of research studies on micro-leakage, the dye-tracer penetration is greater in sites where the cavity margin is in dentin, as compared to the cavities with margins in enamel [6,7]. 

In this study, none of the tested adhesive systems completely eliminated micro-leakage in the tested specimens of dentin margins of the cavity. This is in line with previous studies that investigated the micro-leakage of restoration at the dentin interface [23,24,25]. To overcome this predicament, the present study shows the use of GIC liners (group 4) in the proximal box of class-II preparation has the least leakage among all groups, followed by Bulk-fill with the self-etch adhesive system (group 2b). The result of Fabianelli et al. study concurs with our study’s result was that the open-sandwich technique resulted in significantly low micro-leakage [26]. Using flowable RBCs as a liner under regular RBCs reduces the micro-leakage compared to regular RBCs without a liner but the difference was not significant (*p* = 0.59). This result is in agreement with the Arora et al. study, which concluded that the use of flowable RBCs liners in a proximal box of class-II preparation has been advocated [27]. Bayraktar et al. [28], investigated the clinical performance of one conventional RBC and three bulk-fill RBCs and reported no statistically significant differences between the materials’ performance at baseline and after a one-year follow-up. Similarly, a recently conducted randomized, prospective study compared the clinical performance of bulk-fill RBCs, conventional RBCs, and a highly viscous GIC for class-II restorations. The authors concluded that both bulk-fill and conventional RBCs demonstrated better clinical performance compared to the highly viscous GIC [29]. Radhika et al., [30] recommended the flowable RBCs lining in case of deep class-II cavities due to their ability to form a flexible intermediate layer, that assists in relieving the stresses of polymerization contraction in the material [31,32]. The highest dye-penetration values were noted for Group 2a (Bulk-fill with total-etch adhesive system) and Group 1a (Regular RBCs with total-etch adhesive system), and 1b (Regular RBCs with total-etch adhesive system), that showed a significant prevalence of Score 2.

The dye-penetration analysis from in vitro research is often higher than the in vivo studies [33]. In the present study, using the open-sandwich technique along with hybrid glass-ionomers led to better marginal adaptation at the cervical margins compared to other intermediate layers or RBCs materials [34]. The usage and role of glass-ionomers in micro-leakage control have been investigated and compared widely with various bonding systems. Several studies have demonstrated the superiority of glass-ionomers in controlling micro-leakage [35,36]. The sandwich technique, in which glass-ionomers are used as liners, is a viable method for micro-leakage reduction [37]. One of the possible reasons for decreased micro-leakage is the reduction in the total volume of RBCs, as a result of lesser dimensional changes that may occur during polymerization [38]. It is challenging for the dental adhesive systems to provide an equally strong and effective bond to two different hard structures of teeth. Research studies have shown the bonding to enamel to be more durable, while the bonding to dentin is far more intricate/complicated and can possibly be achieved only if more complicated, and time-consuming techniques are employed [39]. 

The degradation mechanisms which the dentin undergo during the adhesive procedures are not fully known. For ER bonding systems, a major part of degradation is thought to consist of both hydrolysis and enzymatic degradation of the collagen fibrils and the polymerized resin-matrix present in the hybrid layer [40]. While in the SE adhesives, the impregnation/demineralization of the adhesives into the enamel-dentin support perform concurrently. The major demineralization occurs from the exposure to acidic monomers that are components of most of the adhesive systems. Therefore, it is suggested to avoid rinsing the SE adhesives [41]. SE bonds are believed to have certain benefits including decreased postoperative sensitivity and pain by removal of smear and covering more dentinal tubules than ER bonds [42]. In the present study, the two-step self-etch adhesive system shows superior result in dentin sealing than the total-etch system, this is in agreement with Ozer F, who recommended the use of self-etch adhesive in dentin [43]. 

The in vitro design of the study was the main limitation. Ideally, the micro-leakage should have been tested with variables such as masticatory forces, types of food, oral temperature, moisture, and presence of saliva with enzymes and bacterial by-products. In addition, the study was performed on extracted teeth that could have been affected by demineralization/dryness after the extraction and/or the teeth may have been inherited/acquired with existing micro defects which were not visible visually. Furthermore, in the present research, only vertical sectioning was carried out in the mesial and distal directions. For future studies, it is suggested to employ a more accurate way for the evaluation of the total micro-leakage, that is, complete removal of the restoration and evaluation of the total amount of micro-leakage, that varies in different areas. Furthermore, subjecting the samples to mechanical loading for simulating the intraoral conditions is also suggested for future investigations. 

## 5. Conclusions

Using GIC base in the proximal box of class-II preparation has the best ability to achieve the marginal seal and minimal leakage among all groups, followed by Bulk-fill RBCs using the self-etch adhesive system. The use of a self-adhesive system exhibited superior performance in reducing the micro-leakage than the total-etch adhesive system.

## Figures and Tables

**Table 1:** Details of various restorative materials used in the study.

Material	Manufacturer	Description
Tetric N-Ceram	Ivoclar Vivadent, Schaan, Liechtenstein	Radiopaque nano-hybrid RBCs based on nano-optimized technology that can be light-cured for direct dental restoration.
Tetric N-Flow	Ivoclar Vivadent, Schaan, Liechtenstein	Radiopaque, flowable nano-hybrid RBCs based on nano-optimized technology that can be light-cured for direct dental restoration.
SonicFill™ 2	Kerr Corp., Orange, CA, USA	Resin based direct restorative RBCs with low shrinkage and can be activated using the SonicFill Handpiece (sonically activated delivery).
Fuji II LC	GC, Tokyo, Japan	A dual cure, glass-ionomers restorative featuring high resistance to water which can be finished

**Table 2:** Description of various bonding processes employed in the study.

Bonding System	Used with	Bonding Process
Etch-Rite™ (total-etch)	Tetric N-Ceram Tetric N-Flow SonicFill™ 2	Following preparation, cavity was washed using water, air dried, acid-etching (15 s), and followed by rinsing using water spray, and removal of excess moisture. Using an application brush, the bonding agent (Etch-Rite™ or Obtibond ™ S) was applied to the enamel and dentin followed by air thinning and light curing for 10 s.
Obtibond ™ S (total-etch)	Tetric N-Ceram Tetric N-Flow SonicFill™ 2	
OptiBond™ XTR (self-etch)	Tetric N-Ceram Tetric N-Flow SonicFill™ 2	Following preparation, cavity was washed using water and air dried, OptiBond XTR PRIMER was applied to the enamel/dentin surface using the disposable applicator brush. Scrub the surface with a brushing motion for 20 s, then air thin (5 s) using gentle air pressure. OptiBond XTR ADHESIVE was applied to the enamel/dentin surface using light brushing movements for 15 s, then air thin for 5 s. Light cure for 10 s
Ketac™ Conditioner (3M ESPE, St. Paul, MN, USA)	Fuji II LC (GC, Tokyo, Japan), resin-modified GIC	Following preparation, cavity was washed using water and air dried, conditioner was applied disposable brush to the smear layer, for 10 s. Then rinse with copious amounts of water, and excess moisture was removed.

**Table 3:** The mean micro-leakage score for the total-etch (ER) and self-etch (SE) adhesive systems.

	Total-Etch	Self-Etch
Mean	2.80	2.07
Std. Deviation	1.648	1.388

**Table 4:** Mann–Whitney test for comparing the micro-leakage between the groups (*p* < 0.05).

Groups	1a	1b	2a	2b	3a	3b	4
1a		0.496	0.755	0.049 *	0.596	0.216	0.036 *
1b	0.496		0.329	0.010 *	0.843	0.602	0.005 *
2a	0.755	0.329		0.048 *	0.405	0.126	0.035 *
2b	0.049 *	0.010 *	0.048 *		0.022	0.095	0.399
3a	0.596	0.843	0.405	0.022 *		0.497	0.016 *
3b	0.216	0.602	0.126	0.095	0.497		0.089
4	0.036 *	0.005 *	0.035 *	0.399	0.016 *	0.089	

* Bold numbers means significant difference. The fractions represent the *p*-value and significance difference between study groups.

## Data Availability

The data presented in this study are available on request from the corresponding author.
